# In vitro and in vivo effectiveness of essential oils against coccidia in Black Livorno chickens

**DOI:** 10.1186/s12917-025-04916-4

**Published:** 2025-07-14

**Authors:** Alessia Zoroaster, Marta Raffaelli, Manuela Diaferia, Fabrizia Veronesi, Margherita Marzoni Fecia di Cossato, Barbara Contiero, Roberto Marani, Antonio Frangipane di Regalbono, Stefania Perrucci

**Affiliations:** 1 Department of Animal Medicine, Production and Health, University of Padova, Viale dell’Università 16, Legnaro (Agripolis), Padova, 35020 Italy; 2 Department of Veterinary Sciences, University of Pisa, Viale Delle Piagge 2, Pisa, 56124 Italy; 3 Department of Veterinary Medicine, University of Perugia, Via San Costanzo 4, Perugia, 06126 Italy; 4 Interdepartmental Research Center “Nutraceuticals and Food for Health”, University of Pisa, Via Del Borghetto 80, Pisa, 56124 Italy; 5 La Veterinaria s.r.l., Via A. Einstein 6/8/10, Ponte San Giovanni, Perugia, 06135 Italy

**Keywords:** *Eimeria*, Coccidiosis, Essential oils, Poultry, Local breed, In vitro efficacy, In vivo efficacy

## Abstract

**Background:**

Coccidiosis remains a major challenge in poultry farming due to the drug-resistance phenomena in *Eimeria* strains and the possible risk of accumulation of anticoccidial residues in poultry-derived products. Essential oils (EOs) and their bioactive constituents are being considered for their potential role as alternative control strategies. The present study evaluated the in vitro efficacy of thymol, carvacrol, cinnamic aldehyde, eugenol, and a commercial EO blend (namely Energy Poultry, EP) against *Eimeria* spp., as well as the in vivo efficacy of thymol and EP (supplemented at 5 mg/kg and 50 mg/kg of feed, respectively) in growing chickens.

**Results:**

In vitro assays demonstrated that thymol and carvacrol significantly disrupted oocyst integrity and inhibited sporulation at concentrations ≥ 2%, with maximal degeneration rates of 96% and 90%, respectively, at 10%. Cinnamic aldehyde reduced sporulation by 79%, whereas eugenol showed minimal efficacy. The potential synergistic effect between carvacrol and cinnamic aldehyde of EP resulted in a marked reduction in oocyst viability (~ 90%). Based on these findings, thymol and EP were selected for in vivo evaluation in a native Italian egg-type chicken breed, the Black Livorno. Despite not significant, dietary supplementation (thymol: 5 mg/kg; EP: 50 mg/kg) led to a reduction in oocyst per gram (OPG) values from day 78 onward compared to the control group in which the highest oocyst excretion persisting for an extended period. Growth performance (average daily gain and feed conversion ratio) was not affected, indicating no adverse effects of thymol and EP supplementation. Despite the presence of highly pathogenic *Eimeria* species (*E. tenella*, *E. necatrix*), no clinical signs were observed, probably due to the possible low parasitic burden and breed’s inherent resistance.

**Conclusions:**

The obtained findings suggest that while evaluated EO constituents exhibited promising anticoccidial effects in vitro, their in vivo efficacy may be dose-dependent, influenced by infection pressure and host factors. Further research is warranted to optimise tested EOs inclusion levels and assess their long-term impact on coccidiosis control in poultry production systems.

## Background

Animal proteins represent some of the most valuable and highly sought-after nutritional resources on a global scale. Among these, poultry, particularly chicken, represents a preferred source of animal protein in many regions worldwide due to cost efficiency and widespread acceptance across various religious and cultural contexts [1, 2]. Furthermore, the reduced levels of fat, cholesterol, and sodium, as well as its perceived health benefits, established favoured option on chicken meat amid consumers [2]. The rising demand for poultry meat has prompted ongoing genetic enhancements aimed at producing fast-growing chickens, introducing physiological changes, metabolic disorders, compromised immune systems, and greater vulnerability to infectious diseases [3]. Among these, coccidiosis, a parasitic disease caused by apicomplexan protozoa of the genus *Eimeria*, is considered one of the most important diseases in chickens, affecting the health and wellbeing of infected birds and having a high economic impact on the poultry industry worldwide [4, 5]. The global poultry sector incurs an estimated annual cost from USD 7 billion to USD 13 billion due to coccidiosis [6]. *Eimeria* spp. infection damages host intestinal mucosa, increasing permeability and nutrient loss, impairing digestion and absorption, and causing intestinal inflammation of different severity leading to reduced growth, poor feed conversion and high morbidity or death [7]. Maintaining current levels of poultry production relies heavily on the implementation of effective prophylactic strategies. Existing methods focus mainly on the use of anticoccidial drugs, vaccines, supplemented by improved hygiene and farm management practices [8]. Nevertheless, the abuse of anticoccidials has led to widespread resistance of *Eimeria* spp. across all compounds used for chickens [7]. The accumulation of chemical residues in poultry tissues poses potential health risks to consumers, highlighting the need for alternative approaches [9, 10]. Moreover, the extensive administration of vaccines is restricted by their high cost, and the absence of specific immunological assays to accurately predict their protective efficacy against coccidiosis limiting their applicability [11]. In this context, natural therapies and supplements, including organic acids, minerals, vitamins, probiotics, plant extracts such as essential oils (EOs), amino acids, dietary nucleotides, feed enzymes, and yeast derivatives, may offer safer sustainable strategies for preventing and managing avian coccidiosis [12]. Among these, EOs and their main components, including phenolic compounds, terpenes, and aldehydes, are known for their antibacterial properties, ability to promote gut health, and antiparasitic effects [13, 14]. Novel compounds are typically evaluated through in vivo trials, which are often costly, time-consuming, and sacrifice numerous animals; thus, adopting prior in vitro screening methods for identifying anticoccidial agents could enhance efficiency and ethical standards in research [15]. In the present study, the in vivo anticoccidial efficacy of thymol, a major constituent of some EOs, and an EO blend (Energy Poultry, EP), incorporated into the diet of native Livorno breed chickens, was assessed against a specific *Eimeria* population identified via PCR analysis, following prior in vitro screening of a panel of EO constituents and the EO blend tested for their anticoccidial activities.

## Results

### In vitro experiment

Results on sporulation rates, degeneration, and structural disruptions of oocysts/treatment compared to the controls are reported in Table 1. The tested constituents and EP exhibited limited effectiveness at lower concentrations (0.1%, 1%, 2%), with significant (*p*-value < 0.05) anticoccidial activity starting at concentrations of 5%. Cinnamic aldehyde demonstrated marked efficacy at 5% and 10%, resulting in a substantial reduction in oocyst sporulation rates (59–79%) and significant degeneration (> 50%) (*p*-value < 0.05), characterised by pronounced structural disruptions of both the oocyst and sporocyst walls. Carvacrol exhibited observable oocyst alterations starting at a 2% concentration, though sporulation rates remained high (> 70%). At 5%, a significant (*p*-value < 0.05) decrease in sporulation was recorded (> 50%), while at 10% it induced deformation in nearly 90% of oocysts. Thymol caused a sporulation inhibition significantly higher (*p*-value < 0.05) than other constituents starting at 2%. At 5%, it effectively suppressed sporulation and promoted oocyst degeneration, achieving near-complete efficacy (> 95%) at 10%. Eugenol showed minimal efficacy across most tested concentrations, except at 10%, where it achieved moderate reductions in sporulation and oocyst degeneration, though its overall performance was inferior to other compounds. The commercial blend EP, exhibited substantial anticoccidial effects, reducing sporulation rate, and increasing the percentage of oocyst deformation to 87% and 90%, at concentrations of 5% and 10%, respectively.

**Table 1:** Percentages of in vitro sporulated (S), unsporulated (U), and degenerated (D) oocysts compared to the controls (S = 88.3%; U = 9.0%=; D = 2.7%) at different concentrations/treatment

	Concentrations (%)														
	0.1			1.0			2.0			5.0			10.0		
	S	U	D	S	U	D	S	U	D	S	U	D	S	U	D
Cinnamic aldehyde	94.7	3.7	1.7	89.7	4.7	5.7	90.7	4.0	5.3	41.3*	5.0	53.7*	21.3*	13.0	65.7*
Carvacrol	93.3	6.0	2.7	87.0	8.3	4.7	71.3	9.6	17.3*	46.3*	10.3	43.3*	10.0*	0.0*	88.3*
Thymol	87.3	9.0	6.6	76.0	13.0	11.0*	55.3*	16.0	28.7*	12.7*	2.3*	85.0*	3.7*	0.3	96.0*
Eugenol	92.3	6.0	1.7	91.3	5.3	3.3	90.0	9.0	1.0	79.0	8.3	10.0	44.3*	13.0	42.7*
Energy Poultry (EP)	87.3	9.0	3.7	88.3	9.0	2.3	89.3	7.3	3.3	10.7*	1.7	87.7*	7.3*	2.3	90.7*

* Statistical significance (*p* < 0.05) compared to the control

### In vivo experiment

**Fig. 1:**
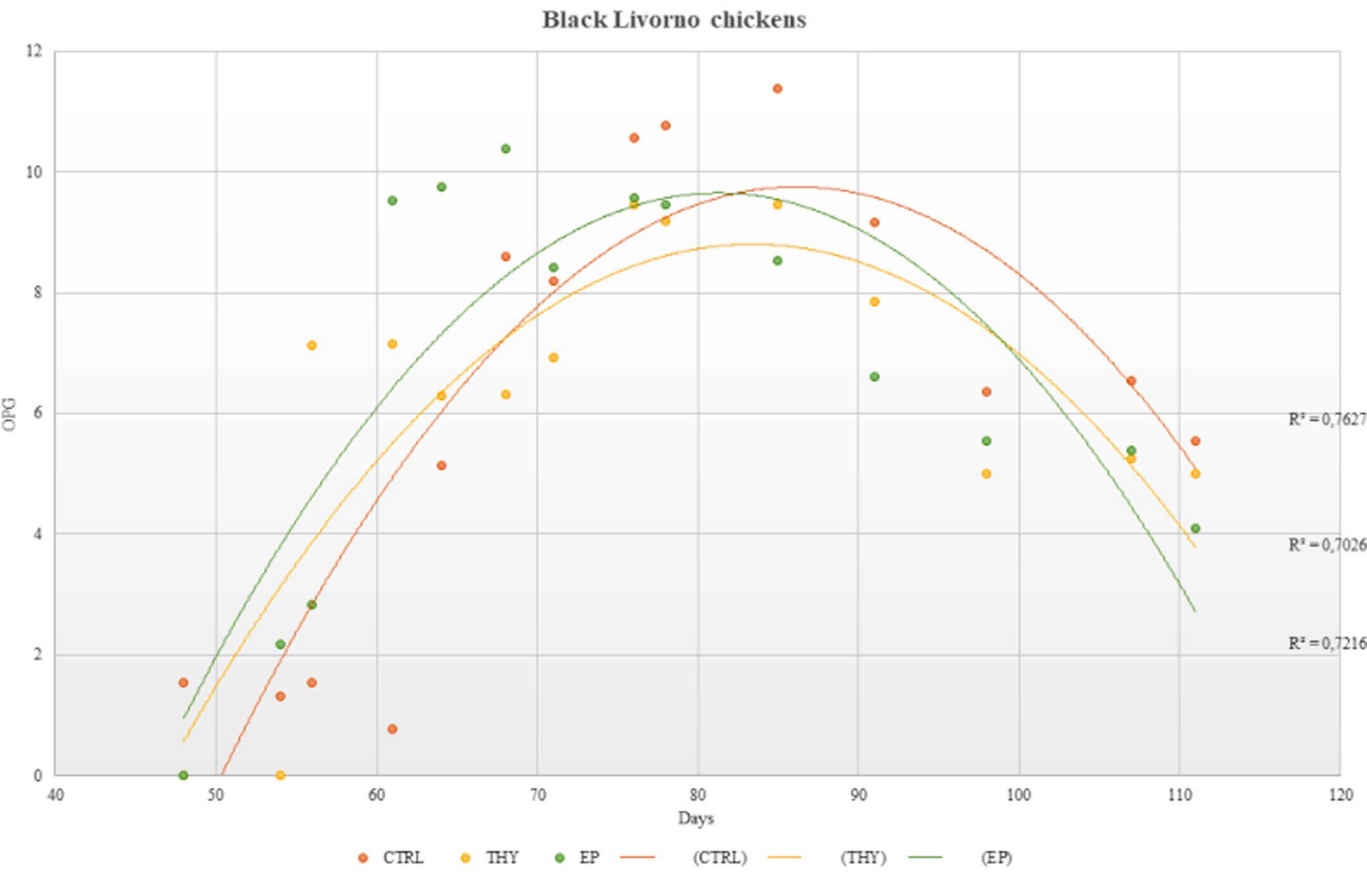
Time course of *Eimeria* spp. oocysts per gram of faeces (OPG) recorded throughout the 110-day trial period in Black Livorno chickens. Data are presented as the log-transformed mean ± SEM from three replicates/treatment

No clinical signs referred to coccidian infection were observed in the whole study period. Observed mortality was: 2 birds in EP, 2 birds in control, and 1 bird in thymol groups. No oocysts were detected until day 40 of age. A slight increment in oocysts shedding was noted from days 56–64 across all experimental groups. Pooled faecal samples were examined for PCR analysis starting on day 64, coinciding with a marked increase in oocysts shedding, with mean OPG values of 8,308, 8,742, and 53,550 for thymol, control and EP, respectively. The highest mean oocysts shedding was recorded in the control group from day 78 (61,583 OPG), peaking at day 85 (94,000 OPG), before declining from day 98 (below 1,000 OPG) (Fig. 1). Compared to the thymol and EP treatment groups where OPG values began to decline from day 78, the controls exhibited prolonged high oocyst excretion. A total of 9 PCR analyses (3 for each treatment group) were conducted on days 64, 68, and 76 of age. The coccidia species identified on the farm included *Eimeria necatrix*, *Eimeria praecox*, and *Eimeria tenella*, with a percentage identity of 98%, 97% and 99%, respectively, compared with those available in GenBank. Molecular analyses confirmed the presence of *E. praecox* in all treatment groups. Additionally, *E. tenella* was detected in one PCR assay from the control group, while *E. necatrix* was identified in one PCR assay from the thymol-treated group. The comparative analysis of the three dietary treatments (control, thymol-supplemented, and EP-supplemented diets) revealed no statistically significant differences in oocyst count reduction across intra- and inter-time-diet interactions.

In Table 2 are shown the data from growth performance evaluation. Statistical analysis indicated that only the effect of sex was significant for the whole period on average daily weight gain (ADG) (11.1 ± 1.14 g/d for females vs. 14.5 ± 1.2 g/d for males). Within-sex analyses revealed no significant differences of ADG and feed conversion ratio (FCR) among the treatment groups.

**Table 2:** Effects of diet supplementation with thymol and Energy Poultry (EP) on average daily weight gain (ADG) and feed conversion ratio (FCR) of Black Livorno chickens naturally infected by *Eimeria* spp.^a^

	Females			SEM^b^	*P*-value		Males			SEM^b^	*P*-value
	ADG (g)						ADG (g)				
Days of age	Control	EP	Thymol				Control	EP	Thymol		
	(*n* = 14)	(*n* = 13)	(*n* = 15)				(*n* = 14)	(*n* = 16)	(*n* = 12)		
0–28 d	7.06	7.69	7.47	0.45	0.629		7.45	8.15	8.44	0.47	0.346
28–110 d	11.08	9.68	10.57	0.43	0.129		15.80	16.12	15.91	0.53	0.888
0-110 d	10.47	10.25	10.76	0.30	0.483		14.37	14.28	14.55	0.90	0.894
			**FCR**								
			**Days of age**	**Control**	**EP**	**Thymol**	**SEM** ^b^	***P-value***			
				(***n* = 28**)	(***n* = 29**)	(***n* = 27**)					
			0–28 d	2.96	2.88	3.02	0.15	0.825			
			28–110 d	6.84	6.62	6.32	0.42	0.692			
			0-110 d	5.55	5.35	5.22	0.27	0.702			

^a^ ADG values represent the mean of individual male and female ADG measurements, averaged over three replicates per treatment. FCR values were calculated based on pen-level data, with three replicates per treatment; ^b^ SEM= standard error of the means

## Discussion

The control of chicken coccidiosis remains a critical challenge due to the cost of vaccination, the widespread emergence of drug-resistant *Eimeria* strains, and the potential accumulation of anticoccidial residues in poultry products, posing significant implications for public health and food safety [16, 17]. Therefore, the development of alternative/complementary control strategies that ensure effective disease management are needed. The inclusion of EOs as dietary supplements has gained importance due to their potential to enhance and optimise performance parameters and support gut health [18]. EOs constituents may represent a promising approach to conventional antimicrobials, mitigating resistance phenomena and enhancing a sustainable and efficient production system. In the present study, the EO-derived compounds thymol, carvacrol, cinnamic aldehyde, and EP were found to inhibit the sporulation and/or alter the oocysts of chicken *Eimeria* spp. in vitro at the highest concentrations tested. Compared to the controls, the effectiveness of the constituents tested in vitro was markedly diminished at lower concentrations (0.1%, 1%, 2). However, effects became more pronounced and potentially applicable for practical use at concentrations of 5% or higher. Nevertheless, carvacrol and thymol exhibited noteworthy bioactivity starting at a concentration of 2%. Carvacrol significantly disrupted oocyst integrity, although the sporulation rate remained elevated. Conversely, thymol demonstrated higher sporulation inhibition efficacy compared to other compounds tested at the same concentration. At a concentration of 10%, both these compounds induced substantial oocyst degeneration, reaching 90% and 96%, respectively. At the same concentration, cinnamic aldehyde reduced oocyst sporulation by 79%, whereas the effects observed with eugenol were minimal, even at this high concentration. Instead, the potential synergistic interaction between cinnamic aldehyde and carvacrol, two key constituents of the commercial EO blend EP, may have contributed to the significant reduction in oocyst sporulation and degeneration, approaching 90%. A comparable in vitro study conducted by [19] reported that, among the ten EOs tested, thyme, rich in thymol and carvacrol as its major constituents, demonstrated the highest efficacy in destroying *Eimeria* oocysts. Moreover, the ability of a *Trachyspermum ammi* plant extract in stopping the sporulation process and damaging the morphology of chicken *Eimeria* oocysts in dose dependent manner was referred to its content of thymol and carvacrol bioactive compounds [20]. Using an in vitro cell model, such as Madin-Darby Bovine Kidney [21], showed that thymol- and carvacrol-based formulations exhibited the highest efficacy in inhibiting the invasion of *Eimeria* sporozoites at 2 h *post*-infection, with a potential in vivo application. Thus, the activity of these compounds could be attributed to the hydrophobic properties and low molecular weight of EO components, which can disturb the integrity and increase the permeability of both oocyst and sporocyst walls, leading to dehydration and resulting in structural deformation and a loss of the ability for the oocyst to sporulate and acquire infectivity [22]. Our findings in vitro underscore the potential of EO major constituents as promising agents for controlling coccidian infections in poultry [23]. The findings described above permitted to select EP and thymol for the in vivo evaluation. Dietary supplementation with these EO constituents (50 mg/kg and 5 mg/kg, respectively) resulted in a reduction in OPG values from day 78 of age compared to the controls, in which higher mean oocyst excretions persisted for an extended period. Nevertheless, the decline did not correlate with a statistically significant reduction in oocyst shedding, unlike the pronounced effects observed in vitro. These outcomes are consistent with data from a previous study in which no significant differences were observed in the oocysts count (OPG) of broilers fed with diets containing thymol and carvacrol at various concentrations, compared to the controls [24]. However, it should also be considered that the concentrations of tested constituents in the diet used in the present and other studies may have been insufficient to effectively reduce oocyst shedding, considering that the inclusion of higher concentrations of thymol and carvacrol in poultry diets has proven successful in other studies [25, 26]. Usually, the severity of avian coccidiosis is closely associated with the *Eimeria* species involved and the number of oocysts ingested by the host [27, 28]. In our study, the absence of clinical signs, including the low mortality rate, in all tested birds is notable given that molecular analysis identified the presence of at least two highly pathogenic *Eimeria* species on the farm, i.e., *E. tenella* and *E. necatrix* [29],. Although OPG values can fluctuate over time due to variations in the life cycle of chicken *Eimeria* spp [7]., the parasitic load on the farm may presumably be regarded as relatively low [4]. However, the absence of symptoms in the present study could be attributed to the use of a native chicken breed, which some of them are known for a greater resistance to coccidiosis compared to commercial breeds [30]. These findings highlighted the importance of considering infection pressure, baseline parasite loads, and breeds when evaluating the efficacy of dietary strategies for coccidiosis control in field research. Despite the slight in vivo anticoccidial efficacy observed in this study, a multidisciplinary approach integrating parasitological evaluation and performance indicators (e.g., body weight BW, ADG, FCR) is essential for comprehensively assessing the impact, given the commercial importance of these animals. Growth performance data obtained in this study showed no differences between treatment and control groups, suggesting that diet supplements with thymol and EP had no detrimental effects on Black Livorno birds.

## Conclusions

In vitro findings suggested EO-derived constituents such as thymol, carvacrol, and the blend EP as alternative tools for controlling *Eimeria* spp. in poultry, whereas the in vivo effects were modest, underscoring the need for further investigation. Future research should focus on optimizing EO concentrations and formulations to enhance their effectiveness under field conditions, particularly in the control of coccidiosis in intensive poultry farming systems. Additionally, the potential synergistic effects of EO constituents warrant further exploration, particularly in combination to achieve an effective control strategy. Investigating the mechanisms by which EOs disrupt oocyst integrity, including their hydrophobic properties and effects on cellular permeability, could provide valuable insights into developing targeted anticoccidial interventions. Considering breed differences in coccidiosis resistance, it is crucial to integrate studies on native chicken breeds alongside commercial strains to better understand the role of genetic resistance in the efficacy of dietary supplementation with EO-derived constituents. Furthermore, addressing the variations in parasitic load, infection pressure, and environmental factors is important for translating in vitro outcomes into routine applications. Given the concerns surrounding drug resistance and the public health implications of anticoccidial residues, there is a growing need to explore sustainable and cost-effective alternatives. Further clinical trials, long-term studies, and evaluations of thymol and EP toxicity along with histological evaluations and scoring of intestinal lesions in treated and untreated chickens infected by coccidia, are necessary to determine their safety and effectiveness at commercial scale. A comprehensive approach that combines parasitological evaluations with performance metrics such as growth rate and feed conversion efficiency will provide a more holistic understanding of EOs’ role in poultry health management.

## Methods

Energy Poultry (EP) and a selected panel of EO constituents were first evaluated through in vitro assays. EP is a formulation comprising 47.9% cinnamaldehyde, 45% silicic acid, 5% xanthan gum, 1% eucalyptol aroma, 1% eugenol, and 0.1% carvacrol. Thymol and EO derived constituents present in EP, i.e. cinnamaldehyde, carvacrol, and eugenol, were used at 99% purity. All tested compounds were sourced from La Veterinaria s.r.l., Ponte San Giovanni (PG), Italy.

### In vitro assays

For the in vitro assay, pooled samples collected from naturally infected chickens obtained across various farms situated in Umbria (Perugia), central Italy, were used. Prior parasitological analyses revealed a significant presence of *Eimeria tenella*,* Eimeria maxima*,* Eimeria acervulina*,* Eimeria necatrix*, and *Eimeria mitis* in these samples (unpublished data). Oocysts were harvested and concentrated via flotation in a saturated NaCl solution (specific gravity 1.2) and rinsed three times in distilled water by centrifugation (1,900 rpm for 4 min) to remove salt residues. After counting using the modified McMaster technique (sensitivity of 50 oocysts per gram of faeces, OPG), the oocysts suspension was standardized to approximately 10⁵ unsporulated oocysts/ml. Oocysts were then stored at + 4 °C to preserve viability.

For the in vitro evaluation, EP and pure constituents were emulsified in a solution containing 5% ethanol and 5% Tween 80, diluted in 0.9% NaCl saline solution, resulting in five concentrations (0.1%, 1%, 2%, 5%, and 10%) for each compound tested. One ml of each concentration was added to the wells of 6-well cell culture plates containing 2 ml of a suspension with 10⁵ unsporulated oocysts. Wells added with 5% ethanol and 5% Tween 80 solution served as controls. Further controls, consisting of 10⁵ oocysts in distilled water, were also included. The assay was conducted in triplicate. The plates were incubated at room temperature for 48 h, and the oocysts were isolated by performing three cycles of washing and centrifugation (1,900 rpm for 4 min) in distilled water, collecting the obtained pellet. Afterwards, the purified oocysts were suspended in a 2.5% potassium dichromate solution for 72 h to induce sporulation, followed by collection through centrifugation using the same settings, and subsequent evaluation of the anticoccidial activity of tested compounds. Efficacy was assessed under a microscope at 400X magnification by evaluating reduction in sporulation rates, degeneration, and structural disruptions of 100 oocysts (and their sporocysts)/treatment compared to the controls.

### In vivo experiment

#### Animals, management, and diets

The study was conducted without an experimental *Eimeria* spp. challenge. During the trial, birds became infected under natural exposure conditions. This approach was chosen to better reflect typical field situations.

The trial involved 84 Black Livorno chickens (*Gallus gallus domesticus*), an Italian native slow-growing breed, of both sexes, hatched in March 2024. Each bird underwent individual identification via wing tags. All animals were vaccinated against pseudo-poultry plague (Newcastle disease) in accordance with DGVA regulation VIII/29,204/P-I.8.d/158 of 2005. The experimental trial was carried out at the Podere “Le Querciole” poultry facility of the University of Pisa. Three homogeneous groups were established based on weight and diet, with three replicates per treatment, each comprising 8–10 Black Livorno chickens. The experimental design included the following treatments: three replicates were fed a commercial standard diet without supplementation and served as the control group, three replicates were provided the standard diet supplemented with thymol (5 mg/kg), and three replicates received the standard diet supplemented with EP (50 mg/kg). The animals were allocated to the experimental groups in a decreasing yet balanced manner across replicates. In detail, the control group consisted of 10, 9, and 9 birds; the EP group of 10, 10, and 9 birds; and the Thymol group of 10, 9, and 8 birds. One-day-old chicks were housed in metal cages elevated above ground level with wire mesh flooring, with each cage assigned to a specific group and diet. The cages were housed in indoor facilities with controlled environmental conditions for temperature and humidity, starting from 28 to 30 °C and gradually decreasing to 22–24 °C during the first month of life and 55 ± 5% RH, respectively. Chicks were raised on hay litter equipped with infrared lamps (150 W, 301x) installed in the cages for supplemental heat and lighting during the initial two weeks. After the first week, the hay litter was removed. At 30 days of age, under favourable weather conditions, each group was transferred to outdoor pens measuring 4 m², with a stocking density of 2–3 birds/m². The allocation adhered to the experimental design and followed a randomised model to minimise potential biases from factors such as pen location, weather, sunlight exposure, and management practices. The experimental trial lasted 110 days, adhering to species-specific animal welfare guidelines. All diets were prepared and supplied by La Veterinaria s.r.l., Ponte San Giovanni (PG), Italy. The feed was formulated to meet the specific needs of the chicken breed and was provided *ad libitum* throughout the trial. A starter diet was administered during the initial phase until the birds reached 30 days of age, after which a grower diet was introduced and maintained up to the conclusion of the production cycle. The composition of the starter and grower diets is reported in Table 3.

The study received prior approval from the Ethical Committee for Animal Welfare at the University of Pisa (Organismo Preposto al Benessere degli Animali, OPBA), resolution 18/2024 of 19/03/2024, in compliance with Article 2, paragraph 1, letter b) of Legislative Decree 26/2014.

**Table 3:** Ingredients and chemical composition of the standard diets

Starter diet (1–28 d)		Finisher diet (28–110 d)	
Ingredients	(g/kg)	Ingredients	(g/kg)
Corn meal	47.50	Corn meal	43.90
Soybean meal	33.80	Soybean meal	28.00
Corn gluten meal	5.00	Wheat flour	15.00
Hard wheat middling	3.90	Soft wheat middlings	5.00
Dicalcium phosphate	2.60	Beef tallow	2.00
Molasses	2.00	Corn gluten meal	2.0
Soybean oil	2.00	Calcium carbonate	1.2
Calcium carbonate	1.50	Dicalcium phosphate	1.2
Sodium bicarbonate	0.15	Soybean oil	0.4
Calcium gluconate	0.20	Sodium bicarbonate	0.15
Sodium chloride	0.20	Sodium chloride	0.25
Liquid methionine	0.20	Liquid methionine	0.15
Lysine	0.10	Pellet binder	0.1
Calcium butyrate	0.05	Acidifier	0.1
Multigrain	0.1	Multigrain	0.05
Acidifier	0.1		
Pellet binder	0.1		
Mineral-vitamin premix*	0.5	Mineral-vitamin premix*	0.5
Total	100		100
Chemical composition**			
DM, %	88.00	DM	88.00
CP, %	23.00	CP	21.00
EE, %	4.60	EE	5.00
CF, %	3.00	CF	3.20
Ash, %	7.90	Ash	6.00
*Mineral composition*		*Mineral composition*	
Ca, %	1.40	Ca	0.90
P, %	0.84	P	0.60
Na, %	0.15	Na	0.17
*Aminoacids*		*Aminoacids*	
Methionine, %	0.57	Methionine	0.47
Lysine	1.26	Lysine	1.09

* Vitamin and mineral premix provided the flowing nutrients per kg of diets: vitamin A, 10,000.00 IU; vitamin D_3_ 3,000.00 IU; vitamin E 40.00 mg; vitamin K_3_ 2.00 mg; biotin 0.10 mg; niacin 20.00 mg; pantothenic acid 7.5 mg; thiamine 2.0 mg; cobalamin 0.02 mg; riboflavin 6.00 mg; pyridoxine 3.00 mg; Fe 60.00 mg; Mn 75.00 mg; Zn 40.00 mg; Se 0,20 mg; I 1.25 mg; Cu 4.8 mg; ** DM, Dry Matter; CP, Crude Protein; EE, Ethereal Extract; CF, Crude Fiber

### Growth performance

At hatching, at 15 days of age, and subsequently at biweekly intervals, individual body weight (BW) and feed intake (FI) were measured using a digital platform scale placed in a designated room/pen. The average daily weight gain (ADG) and feed conversion ratio (FCR)/pen were calculated accordingly. Mortality was recorded as it occurred.

### Parasitological analyses

The trial included faecal sampling (2 pools/group/diet) at 15 and 30 days of age, followed by weekly collections. From 60 to 81 days of age, sampling frequency increased to twice per week to pinpoint the peak oocyst shedding. After 81 days of age, faecal samples were collected again on a weekly basis, till to day 110. For the detection and quantification of *Eimeria* spp. oocysts, samples were analysed by flotation test and a McMaster technique with a detection limit of 50 OPG [31] with a low-density flotation solution (NaCl saturated solution, 1.2 specific gravity). To assess the *Eimeria* spp. population, oocyst shedding was monitored across all experimental groups from the initial detection through 15 days *post*-detection, to encompass the typical peak shedding window, which occurs between 5- and 7-days *post*-infection [32]. Samples found positive for at least 500 *Eimeria* spp. oocysts/group of treatment were preserved in 2.5% potassium dichromate, then washed three times in distilled water by centrifugation (2,200 rpm for 9 min), and frozen at -20 °C for subsequent molecular analyses.

### DNA extraction from oocyst samples

Aliquots of 200 µl were taken from thawed oocysts and processed for the DNA extraction using the “Biological Fluids & Cells” workflow from the Quick-DNA Miniprep Plus Kit (Zymo Research, Freiburg, Germany), according to the manufacturer’s instructions.

### Identification of *Eimeria* spp. using end-point PCR

Genus-specific primers for *Eimeria* were utilised to amplify the 18S region [33], and the internal transcribed spacers region (ITS1) of the ribosomal RNA [34] (Table 4). PCR amplification protocol for 18S rRNA gene consisted of Taq polymerase (Platinum Taq DNA Polymerase, Invitrogen) activation for 2 min at 94 °C, followed by 12 cycles of denaturation at 94 °C for 30 s, touch-down annealing from 64 °C to 58 °C with a stepwise temperature decrement of 0.5 °C at every cycle for 30 s, and extension at 72 °C for 40 s. Further 28 cycles of denaturation at 94 °C for 30 s, annealing at 58 °C for 30 s and extension at 72 °C for 40 s were performed. A final extension for 1 minute at 72 °C completed the reaction. The thermal protocol designed for species-specific primers targeting the ITS1 region, was conducted with PCR reactions performed in individual tubes under the same conditions as previously described, except for varying annealing temperatures specific to each *Eimeria* species (Table 4). When necessary, to optimise PCR amplifications, 1–2 µl of dimethyl sulfoxide (DMSO) was incorporated into the reaction mix. Each assay included appropriate controls, comprising a no-template negative control prepared with the PCR reaction mixture, and a positive control utilising the Paracox^®^ 8 vaccine (MSD, Animal Health). PCR products were analysed by an electrophoretic run on a SYBR safe stained 2% agarose gel in TBE buffer 1X. PCR amplicons were sequenced by an external service (Macrogen Europe, Milan, Italy) using the same primers. Sequences were determined in both strands, aligned and the obtained consensus was compared with those available on GenBank using the Basic Local Alignment Search Tool (BLAST: https://blast.ncbi.nlm.nih.gov/Blast.cgi, accessed in September 2024).

**Table 4:** Primers used for the identification of *Eimeria* spp. in *Gallus gallus domesticus*

Target	Primer sequence 5’−3’ (forward and reverse)	Annealing temperature (°C)	Expected product size (bp)	Species
18 S rRNA	F-CGCGCAAATTACCCAATGAA	64 − 58*	450	
	R-ATGCCCCCAACTGTCCCTAT			
ITS1	F-AATTTAGTCCATCGCAACCCT	55	278	*Eimeria tenella*
	R-CGAGCGCTCTGCATACGACA			
ITS1	F-TACATCCCAATCTTTGAATCG	50	285	*Eimeria necatrix*
	R-GGCATACTAGCTTCGAGCAAC			
ITS1	F-GGCTTGGATGATGTTTGCTG	54	321	*Eimeria acervulina*
	R-CGAACGCAATAACACACGCT			
ITS1	F-CGTTGTGAGAARACTGRAAGGG	55	145	*Eimeria maxima*
	R-GCGGTTTCATCATCCATCATCG			
ITS1	F-CATCATCGGAATGGCTTTTTGA	50	368	*Eimeria praecox*
	R-AATAAATAGCGCAAAATTAAGCA			
ITS1	F-TATTTCCTGTCGTCGTCTCGC	54	306	*Eimeria mitis*
	R-GTATGCAAGAGAGAATCGGGA			
ITS1	F-GATCAGTTTGAGCAAACCTTCG	55	311	*Eimeria brunetti*
	R-TGGTCTTCCGTACGTCGGAT			

* Touch-down 0.5 °C/cycle

### Statistical analysis

For the in vitro experiment, WinPepi version 11.65 (PEPI-for-Windows, London, UK) was used for statistical analysis. The anticoccidial activity of the tested EO constituents/blend at concentrations of 0.1%, 1%, 2%, 5%, and 10% was determined by comparing the percentage of non-sporulated, sporulated, and degenerated oocysts to that of the controls. An independent two-sample t-test was used for comparisons. For the in vivo experiment, the data related to the recovered oocysts were log-transformed to achieve normality. The effect of time within the three treatment groups and the effect of treatments within different ages were analysed using an ANOVA linear model, SAS 9.3 statistical analysis software for Windows (SAS Institute Inc., Cary, NC). For each animal, ADG was calculated as the regression coefficient of BW related to age during the growth trial. Statistical analysis was conducted using an ANOVA model, with treatment group (control, EP, thymol), replicate (1, 2, 3), sex (male - M, female - F), and their interactions included as fixed effects. A separate ANOVA by sex and period (0–28 d and 28–110 d) was conducted considering the effects of the treatment group and replicate. FCR was also analysed using a one-way ANOVA with treatment group as fixed effect. Post-hoc pairwise comparisons were performed using Bonferroni correction. Data were expressed as the mean ± standard error of the mean (SEM). A *p*-value < 0.05 was considered statistically significant.

## Data Availability

The data sets and materials in the present study are available from the corresponding author upon request.

## References

[1] Lawal RA, Hanotte O. 2021. Domestic chicken diversity: origin, distribution, and adaptation. *Anim. Genet.* 2021; *52*:385–394. 10.1111/age.13091 34060099

[2] Connolly G, Campbell WW. Poultry consumption and human cardiometabolic health-related outcomes: a narrative review. Nutrients. 2023;15:3550. 37630747 10.3390/nu15163550 PMC10459134

[3] Bogucka J, Stadnicka K. Quality of poultry meat-the practical issues and knowledge based solutions. Phys Sci Rev. 2023;8:4415–33.

[4] Haug A, Gjevre AG, Skjerve E, Kaldhusdal M. A survey of the economic impact of subclinical *Eimeria* infections in broiler chickens in Norway. Avian Pathol. 2008;37:333–41. 18568662 10.1080/03079450802050705

[5] Quiroz-Castañeda RE, Dantán-González E. Control of avian coccidiosis: future and present natural alternatives. BioMed Res Int 2015; 430610. 10.1155/2015/430610 PMC4346696 25785269

[6] Taylor J, Walk C, Misiura M, Sorbara JOB, Giannenas I, Kyriazakis I. Quantifying the effect of coccidiosis on broiler performance and infection outcomes in the presence and absence of control methods. Poult Sci. 2022;101:101746. 35219136 10.1016/j.psj.2022.101746 PMC8881651

[7] Andreopoulou M, Chaligiannis I, Sotiraki S, Daugschies A, Bangoura B. Prevalence and molecular detection of *Eimeria* species in different types of poultry in Greece and associated risk factors. Parasitol Res. 2022;121:2051–63. 35499632 10.1007/s00436-022-07525-4

[8] Hafez HM. Poultry coccidiosis: prevention and control approaches. Arch Fur Geflugelkd. 2008;72:2–7.

[9] Saeed Z, Alkheraije KA. Botanicals: a promising approach for controlling cecal coccidiosis in poultry. Front Vet Sci. 2023;10:1157633. 37180056 10.3389/fvets.2023.1157633 PMC10168295

[10] Ahmad R, Yu YH, Hua KF, Chen WJ, Zaborski D, Dybus A, Hsiao FSH, Cheng YH. 2024. Management and control of coccidiosis in poultry - a review. *Anim. Biosci.* 2024; *37*:1–15. 10.5713/ab.23.0189 PMC10766461 37641827

[11] Soutter F, Werling D, Tomley FM, Blake DP. Poultry coccidiosis: design and interpretation of vaccine studies. Front Vet Sci. 2020;7:101. 32175341 10.3389/fvets.2020.00101 PMC7054285

[12] Alsayeqh AF, Abbas RZ. Nutritional supplements for the control of avian coccidiosis – a review. Ann Anim Sci. 2023;23:993–1007.

[13] Idris M, Abbas RZ, Masood S, Rehman T, Farooq U, Babar W, et al. The potential of antioxidant rich essential oils against avian coccidiosis. Worlds Poult Sci J. 2017;73:89–104.

[14] Stevanović ZD, Bošnjak-Neumüller J, Pajić-Lijaković I, Raj J. Vasiljević, M. Essential oils as feed additives—future perspectives. Molecules. 2018;23:1717. 30011894 10.3390/molecules23071717 PMC6100314

[15] Felici M, Tugnoli B, Piva A, Grilli E. In vitro assessment of anticoccidials: methods and molecules. Animals. 2021;11:1962. 34209100 10.3390/ani11071962 PMC8300270

[16] Abbas RZ, Munawar SH, Manzoor Z, Iqbal Z, Khan MN, Saleemi MK, et al. Anticoccidial effects of acetic acid on performance and pathogenic parameters in broiler chickens challenged with *Eimeria Tenella*. Pesqui Vet Bras. 2011;31:99–103.

[17] Chen N, Cai Q, Wang S, Song Q, Xie Y, Shi H, et al. Evaluation of the efficacy of myrcene in the treatment of *Eimeria Tenella* and *Toxoplasma gondii* infection. J Vet Med Sci. 2025;87:32–42. 39567006 10.1292/jvms.24-0397 PMC11735216

[18] Adaszyńska-Skwirzyńska M, Szczerbińska D. Use of essential oils in broiler chicken production–a review. Ann Anim Sci. 2017;17:317–35.

[19] Remmal A, Achahbar S, Bouddine L, Chami N, Chami F. In vitro destruction of Eimeria oocysts by essential oils. Vet Parasitol. 2011;182:121–6. 21726944 10.1016/j.vetpar.2011.06.002

[20] Abbas RZ, Abbas A, Raza MA, Khan MK, Saleemi MK. Saeed. In vitro anticoccidial activity of *Trachyspermum ammi* (Ajwain) extract on oocysts of *Eimeria* species of chicken. Adv Life Sci. 2019;7:44–7.

[21] Felici M, Tugnoli B, Ghiselli F, Massi P, Tosi G, Fiorentini L, et al. In vitro anticoccidial activity of thymol, carvacrol, and saponins. Poult Sci. 2020;99:5350–5. 33142451 10.1016/j.psj.2020.07.035 PMC7647770

[22] Jitviriyanon S, Phanthong P, Lomarat P, Bunyapraphatsara N, Porntrakulpipat S, Paraksa N. In v*itro* study of anti-coccidial activity of essential oils from Indigenous plants against *Eimeria Tenella*. Vet Parasitol. 2016;228:96–102. 27692340 10.1016/j.vetpar.2016.08.020

[23] Upadhaya SD, Cho SH, Chung TK, Kim IH. Anti-coccidial effect of essential oil blends and vitamin D on broiler chickens vaccinated with purified mixture of Coccidian oocyst from *Eimeria Tenella* and *Eimeria maxima*. Poult Sci. 2019;98:2919–26. 30778571 10.3382/ps/pez040

[24] Yu M, Jeon JO, Cho HM, Hong JS, Kim YB, Nawarathne SR, et al. Broiler responses to dietary 3,4,5-trihydroxybenzoic acid and oregano extracts under *Eimeria* challenge conditions. J Anim Sci Technol. 2021;63:1362–75. 34957450 10.5187/jast.2021.e121 PMC8672266

[25] Arafa WM, Abolhadid SM, Moawad A, Abdelaty AS, Moawad UK, Shokier KAM, et al. Thymol efficacy against coccidiosis in pigeon (*Columba Livia domestica*). Prev Vet Med. 2020;176:104914. 32066028 10.1016/j.prevetmed.2020.104914

[26] Giannenas I, Florou-Paneri P, Papazahariadou M, Christaki E, Botsoglou NA, Spais AB. Dietary oregano essential oil supplementation on performance of broilers challenged with *Eimeria Tenella*. Arch Anim Nutr. 2003;57:99–106. 10.1080/0003942031000107299 12866780

[27] Ngongeh LA, Onyeabor A, Nzenwata E, Samson GK. Comparative response of the Nigerian Indigenous and broiler chickens to a field caecal isolate of *Eimeria* oocysts. J Pathog. 2017;2017:2674078. 28523192 10.1155/2017/2674078 PMC5421090

[28] El-Shall N, Abd El-Hack A, Albaqami ME, Khafaga NM, Taha AF, Swelum AE. Phytochemical control of poultry coccidiosis: a review. Poult Sci. 2022;101:101542. 34871985 10.1016/j.psj.2021.101542 PMC8649401

[29] López-Osorio S, Chaparro-Gutiérrez JJ, Gómez-Osorio LM. Overview of poultry *Eimeria* life cycle and host-parasite interactions. Front Vet Sci. 2020;7:384. 32714951 10.3389/fvets.2020.00384 PMC7351014

[30] Boulton K, Nolan MJ, Wu Z, Psifidi A, Riggio V, Harman K, et al. Phenotypic and genetic variation in the response of chickens to *Eimeria Tenella* induced coccidiosis. Genet Sel. 2018;50:1–12. 10.1186/s12711-018-0433-7 PMC6249784 30463512

[31] Haug A, Gjevre AG, Thebo P, Mattsson JG, Kaldhusdal M. Coccidial infections in commercial broilers: epidemiological aspects and comparison of *Eimeria* species identification by morphometric and polymerase chain reaction techniques. Avian Pathol. 2008;37:161–70. 18393094 10.1080/03079450801915130

[32] Chasser KM, Duff AF, Wilson KM, Briggs WN, Latorre JD, Barta JR, et al. Research note: evaluating fecal shedding of oocysts in relation to body weight gain and lesion scores during *Eimeria* infection. Poult Sci. 2020;99:886–92. 32036984 10.1016/j.psj.2019.10.028 PMC7587844

[33] AL-Zarkoushi MMF, AL-Zubaidi MTS. Molecular study of *Eimeria* species in quail birds (*Coturnix coturnix japonica*) in Thi-Qar province, Southern Iraq. Indian J Forensic Med Toxicol. 2022;16:1674–80.

[34] Schwarz RS, Jenkins MC, Klopp S, Miska KB. Genomic analysis of *Eimeria* spp. Populations in relation to performance levels of broiler chicken farms in Arkansas and North Carolina. J Parasitol. 2009;95:871–80. 20049993 10.1645/GE-1898.1

